# Machine learning techniques for the optimization of joint replacements: Application to a short-stem hip implant

**DOI:** 10.1371/journal.pone.0183755

**Published:** 2017-09-05

**Authors:** Myriam Cilla, Edoardo Borgiani, Javier Martínez, Georg N. Duda, Sara Checa

**Affiliations:** 1 Centro Universitario de la Defensa (CUD), Academia General Militar, Zaragoza, Spain; 2 Aragón Institute of Engineering Research (I3A), University of Zaragoza, Zaragoza, Spain; 3 Julius Wolff Institute, Charité - Universitätsmedizin Berlin, Berlin, Germany; 4 Centro Universitario de la Defensa (CUD), Escuela Naval Militar, Marín, Spain; 5 Berlin-Brandenburg Center for Regenerative Therapies, Charité-Universitätsmedizin Berlin, Berlin, Germany; 6 Berlin-Brandenburg School for Regenerative Therapies, Charité-Universitätsmedizin Berlin, Berlin, Germany; Kanazawa University, JAPAN

## Abstract

Today, different implant designs exist in the market; however, there is not a clear understanding of which are the best implant design parameters to achieve mechanical optimal conditions. Therefore, the aim of this project was to investigate if the geometry of a commercial short stem hip prosthesis can be further optimized to reduce stress shielding effects and achieve better short-stemmed implant performance. To reach this aim, the potential of machine learning techniques combined with parametric Finite Element analysis was used. The selected implant geometrical parameters were: total stem length (L), thickness in the lateral (R1) and medial (R2) and the distance between the implant neck and the central stem surface (D). The results show that the total stem length was not the only parameter playing a role in stress shielding. An optimized implant should aim for a decreased stem length and a reduced length of the surface in contact with the bone. The two radiuses that characterize the stem width at the distal cross-section in contact with the bone were less influential in the reduction of stress shielding compared with the other two parameters; but they also play a role where thinner stems present better results.

## 1.- Introduction

The importance of a medical treatment is reflected by the number of procedures carried out per year in a population. Approximately, one million of hip fractures occur worldwide every year. The rate of hip replacements increased by about 25% between 2000 and 2009 [1], and this trend is expected to continue in the next decades due to the ageing population, improving medical care in developing countries and decreasing average age at the first operation [2]. In addition, children and young people, whose life expectancy largely surpasses the mean lifetime of an implant, and therefore, they often requiring a revision surgery [2], represent a portion of these patients.

After total hip replacement, the presence of a rigid stem into the femur substantially alters the mechanical conditions within the bone when compared with the healthy situation. The inserted implant increases the flexural rigidity leading to a decrease of the mechanical stresses and strains within the bone (stress shielding), especially, in the region farthest away from the implant. This reduction of mechanical stresses and strains often leads to a bone resorption response [3,4], and therefore, to loss of bone which decreases implant stability and longevity [5] and complicates a revision surgery [6].

Hip prostheses are subject to continuous research and development with the aim to increase their lifespan, offer a more physiologic replication of normal human anatomy and reduce the likelihood of complications and revision surgery. This is reflected by the large variety of hip prostheses in the market. Amongst all hip implant designs, short stems were developed for the younger population. They have the advantage of being more bone conservative by allowing, for higher neck retention and maintenance of the medial greater trochanter, a more physiological stress transfer to the proximal femur [7]. Although they do lead to a reduction in the amount of stress shielding, compared with a traditional hip implant [8], current designs have not been able to completely eliminate the stress shielding effect [9].

Machine Learning Techniques (MLTs) explores the development of algorithms that can learn from and make predictions of data. These techniques are characterized by complex algorithms that can be trained to reproduce a model behaviour [10]. They have been applied successfully to a high variety of problems and data for prediction tasks [11] in industry [12], electronic [13], space science [14], geology [15,16] or language [17] amongst many others. Within the medical context, these techniques have been also successfully applied to different clinical applications, for instance; diagnosis of breast cancer or melanomas, interpreting electrocardiograms, diagnosis of dementia, cardiovascular diseases or predicting prognosis and survival rates [18–23]. The benefits of introducing MLT into medical analysis have been proven by an increase of diagnostic accuracy, reduction of costs and human resources [24,25]. However, the potential of MLTs to optimize the design of joint implants has never been investigated before. Although shape optimization algorithms different to MLTs have been used to assess the relationship between the stem performance and its design for long stems [26–30], the potential to further optimize short stem implants has never been addressed. The advantages of the use of MLT for the optimization of hip implants are (i) its feedback capacity [31], (ii) the reduction of the computational costs due to its ability to generalize situations which are not previously taught to the MLT [32] and (iii) its capacity of work in combination with different minimization algorithms [32]. Important in all optimitation methods however, it is to define an adequate implant design criteria; e.g. reduced stress shielding.

Within this context, the aim of this project was to investigate if the geometry of a short stem hip prosthesis can be further optimized to reduce stress shielding effects and achieve better short-stemmed implant performance. To reach this aim, the potential of MLTs, such as artificial neural networks (ANNs) and support vector machines (SVMs), combined with Finite Element (FE) analysis was used. Novel optimization approaches based on finite element and machine learning techniques open new and innovative possibilities for the design of hip implants never explored before.

## 2.- Material & methods

### 2.1.—Source data: 3D FE parametric study

A 3D parametric study of the influence of the main hip implant geometric factors on the mechanical strains induced within the femur was carried out. These data were then used to feed and train a MLT that combined with an optimization algorithm allowed us to find the optimal hip implant design. For this purpose, a 3D parametric model of an implanted femur was development with ABAQUS v6.12 (Dassault Systemes, Vélizy-Villacoublay, France) and FE analyses were performed using the non-linear solver.

**Fig 1:**
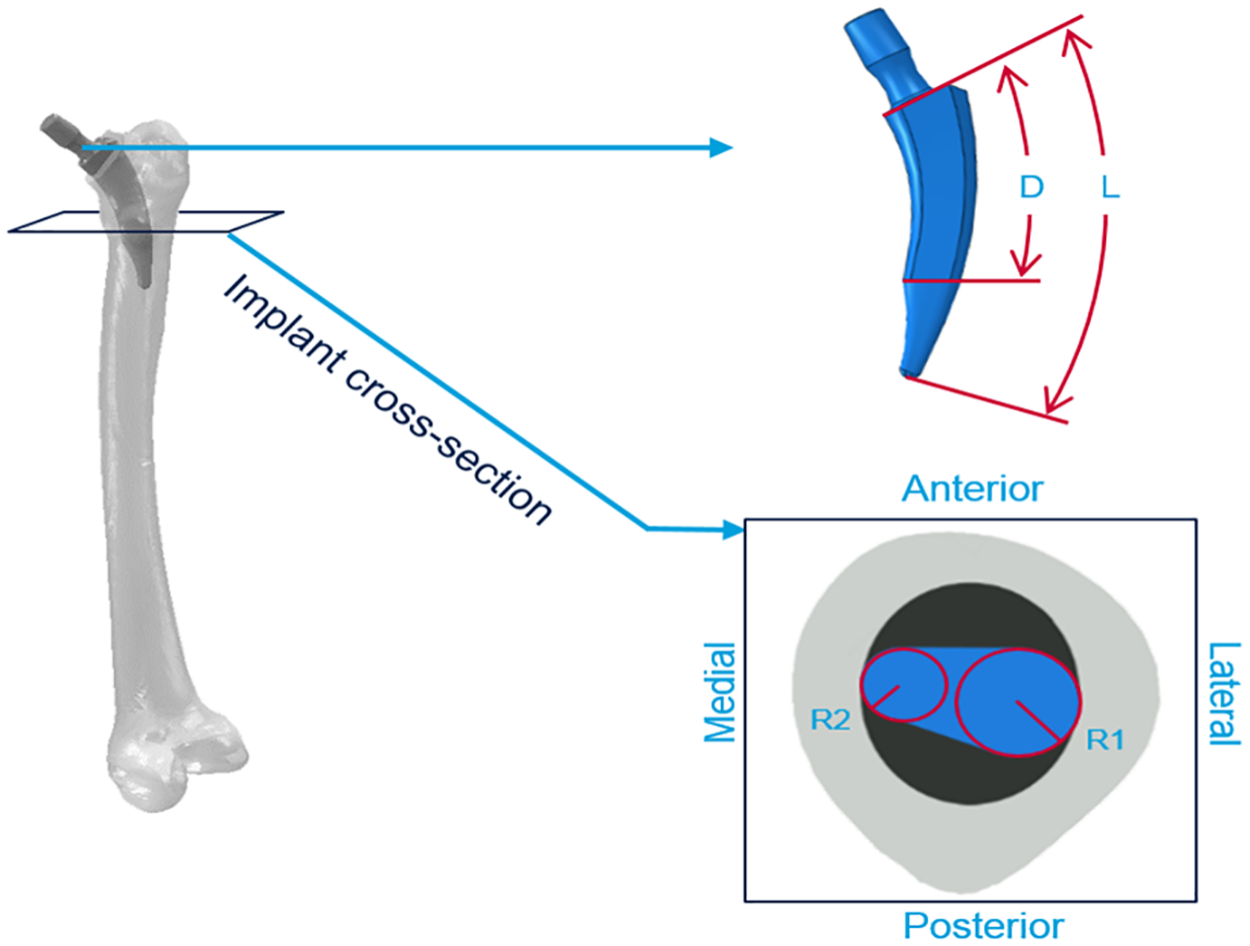
Graphic representation of the four considered parameters. L: total stem length, measured from the implant neck to the distal tip. D: relative distance between the neck and the cross section that separates the part of the stem which is directly in contact with the cortical bone and the part that is not in contact. R1: radius of the circumference in the lateral side of the cross section. R2: radius of the circumference in the medial side of the cross section.

**Hip Implant geometries**: 3D FE models of a short stem implant were developed to determine the influence of the prosthesis on the mechanical strains within the femur. The model is based on an existing short stem implant which is already in the market (Nanos^®^ short stem, Smith & Nephew, Germany) [33,34]. From this implant, different models were computationally created using the same overall geometry but changing four of its dimensions (Fig 1): (1) the total stem length (*L*), measured as the distance from the implant neck to the distal tip, (2) and (3) the stem thickness characterize by the radius in the medial and lateral directions (R1 and R2) of the cross section that separates the part of the stem which is directly in contact with the cortical bone and the part that is not in contact, and 4) the stem-contact-surface internal length (*D*) defined as the distance between the neck and the cross section described in points (2) and (3). This parameter was evaluated as the relative percentage value compared to the stem total length (*L)*.

For each one of these geometrical parameters, realistic data were investigated by varying the total stem length (L: 75, 105, 135 or 165 mm), the radius of the lateral side cross section (R1: 4.5, 5.5, 6.5 or 7.5 mm), the radius of the medial side cross section (R2: 2.0, 2.5, 3.0 or 3.5 mm) and the stem-contact-surface internal length (D: 10, 25, 50 or 75%). Four values for each parameter were considered and combined which resulted in 256 different hip implant models (4^4^ = 256 models). The values were chosen to ensure a clinically admissible shape and taking into account the general dimensions of the selected bone that will host the implant.

Regarding the material properties of the hip implant, titanium alloy was assigned to all implant models and it was considered to be linear elastic, homogeneous and incompressible material with a Young’s modulus of 110.3 GPa and a Poisson's ratio of 0.33 [33].

**Fig 2:**
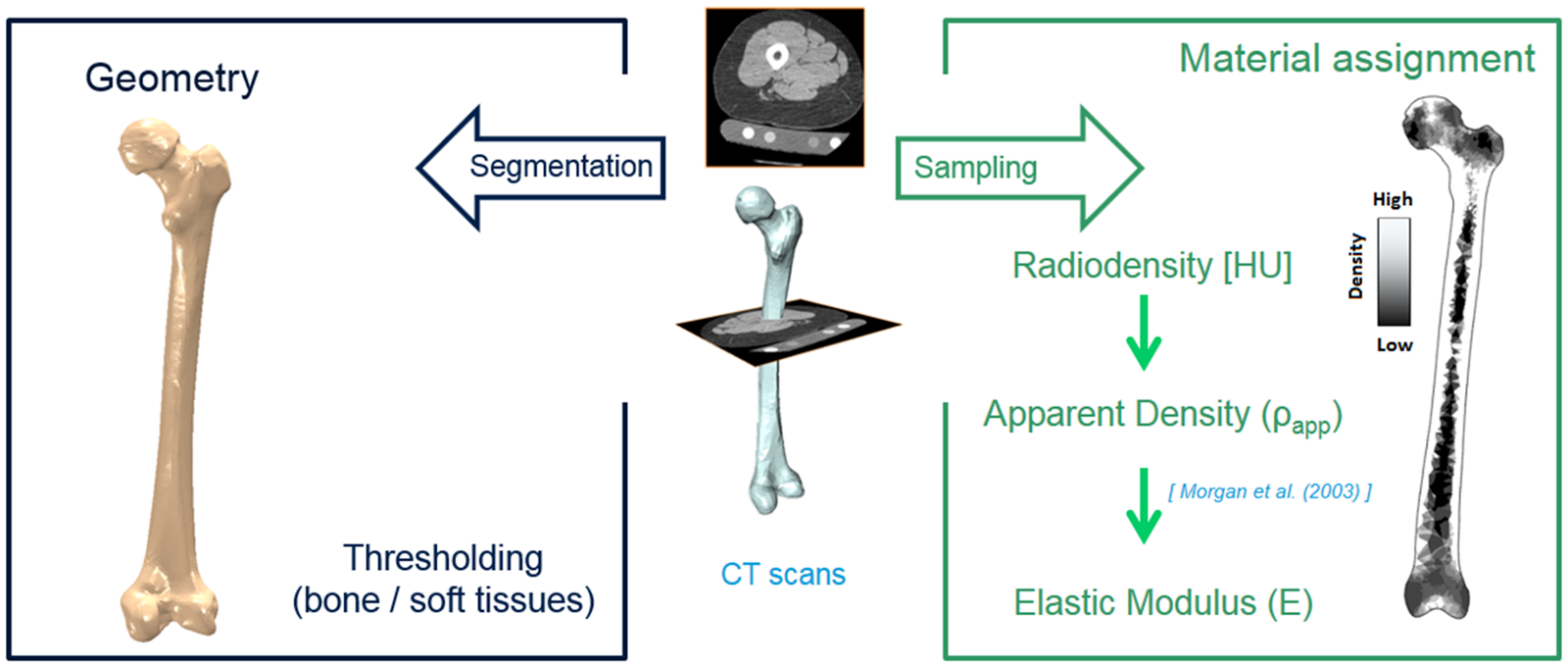
Femur model: Geometry and material properties assignment.

**Bone geometry**: A representative 3D geometry of a right femur was selected out of a larger study of 100 patients who experienced a Total Hip Arthroplasty (THA) in our clinic [35,36] (Fig 2). In that study, proximal bone remodelling was analysed using combined quantitative computed tomography (QTC) and bone remodelling analysis techniques [35,36]. The study was approved by the local ethics committee (Charité ethics board–Approval number: Z5–22462/2–2007–036), and after providing written informed consent to participate, each of the patients received a three joint CT scan (hip, knee, ankle) that included the entire femur, both pre- and post-operatively. The selected bone represented a female patient with mean bone distribution. CT scans were used to segment the femur geometry using ZIBAmira 2013 [37] and Geomagic Studio 10 (3D Systems, Rock Hill, South Carolina, U.S). The same bone geometry was used to generate all the models. CT scans were also used to assign the mechanical properties to each element of the finite element bone model. The scans provided a pixel-by-pixel grey value (radio density) sampling which can be used to estimate the local bone density distribution (Fig 2) [38]. Using the value of the density, the Young’s modulus for each element can be derived using the correlation of Morgan et al. [39].

**Fig 3:**
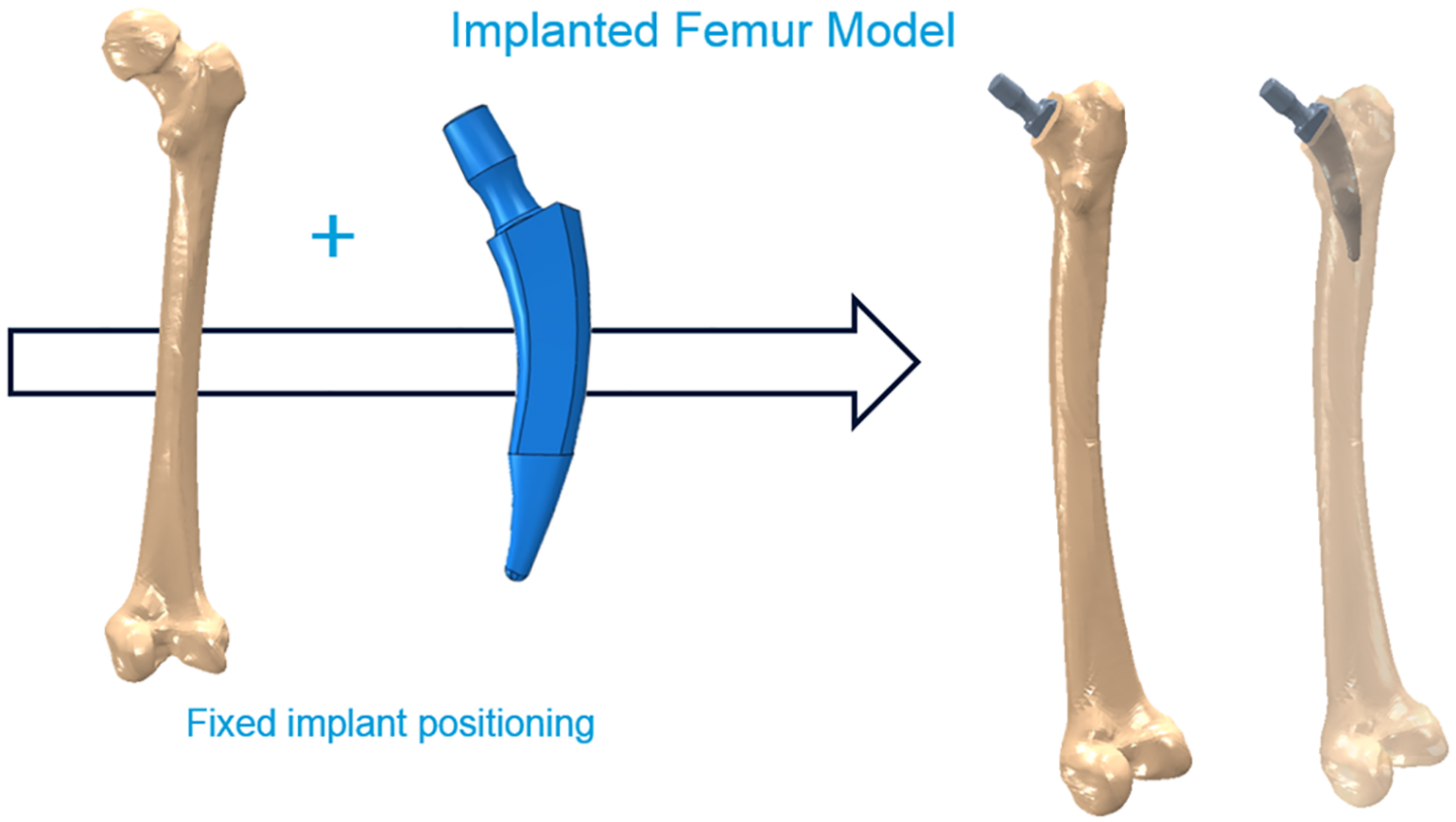
Implanted bone geometry. Hip implant position.

**Implanted bone geometries**: For each of the 256 created implant geometries, an implanted femur model was created using the intact bone geometry (Fig 3). The biomechanical behaviour of the implanted femur with all the different implant designs was then examined and the stress shielding in the proximal region of the femur (Gruen zone I) investigated. The hip joint implant was inserted according to the surgical protocol, which was supervised by our clinical partners. The insertion procedure was the same for all generated models. In addition, for each of the 256 implanted models, a healthy femur model was created maintaining exactly the same mesh as in the implanted femur. This allowed the comparison of implanted and healthy bones in terms of the strain levels in each single element. The difference in absolute maximum principal strain between the implanted and intact models, in each element, was then used to identify the zones where the strains are shielded due to the presence of the implant. Those are the regions where bone resorption is expected.

All the FE models were meshed with ten-nodes tetrahedral elements (C3D10) with a typical edge length of 1.5 mm. Sensitivity analyses were performed to choose the definitive mesh size. To ensure sufficient discretization of the FE models, the element size was decreased until convergence of the predicted strains within the femur was achieved. Convergence was considered when the local strains within the femur did not change more than 5% in subsequent mesh refinement steps.

**Fig 4:**
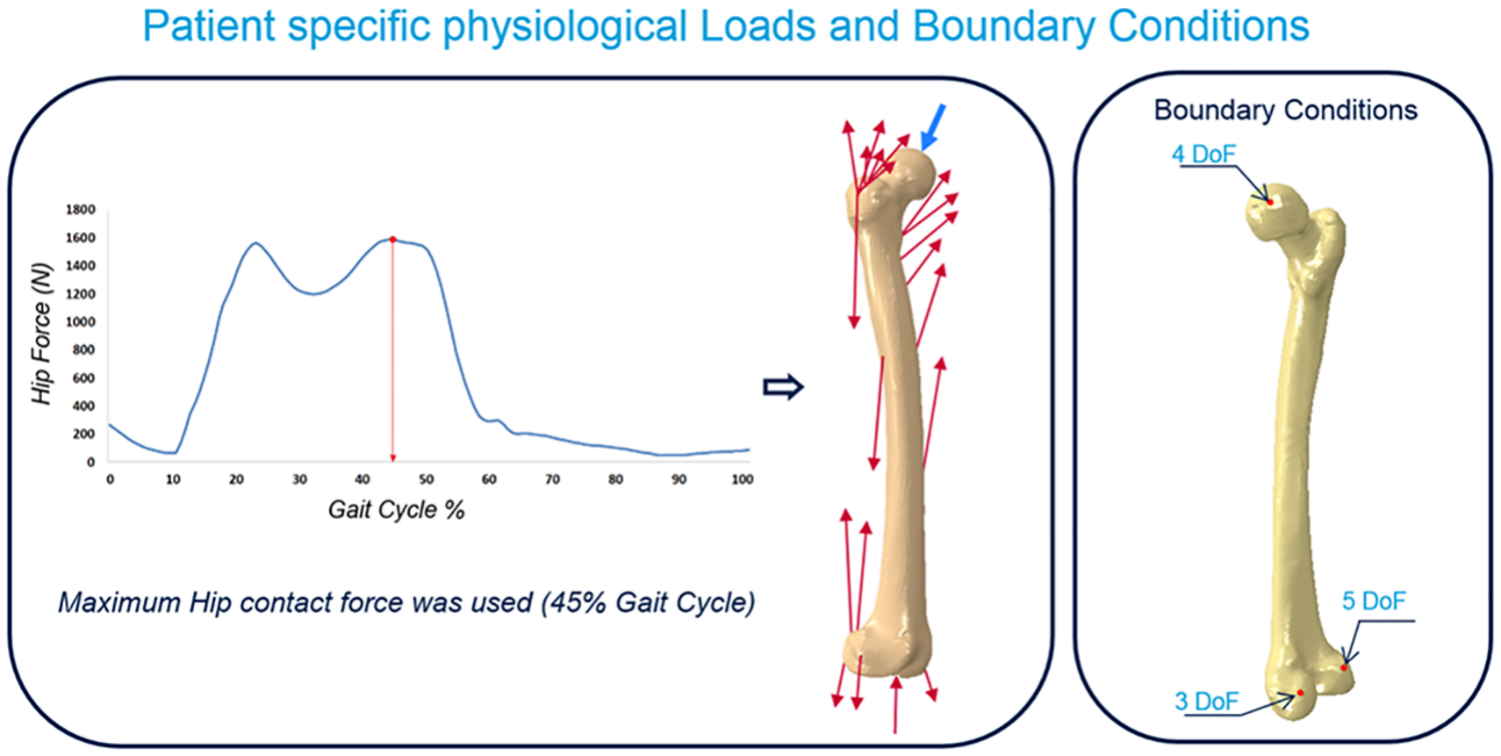
Physiological joint and muscle forces applied to the model and boundary conditions applied to the model.

**Loads and Boundary Conditions**: Physiological loading conditions were obtained using gait analysis and a validated balanced musculoskeletal model [40,41] (Fig 4). Patient-specific muscle and joint contact forces were determined by measuring *in-vivo* movement in a gait analysis laboratory [42]. The patient walked along a gait analysis track equipped with force plates that evaluated the components of ground reaction forces. At the same time, a system of motion-capture cameras (VICON Motion Systems Ltd., Oxford, UK) captured the position of leg landmarks in space to study the movement of the patient lower limb. The anthropometric data, measured directly on our patient, combined with skin markers positioned on specific bone landmarks, allowed us to reconstruct the walking task in a local reference system. Using a musculoskeletal model, joint and muscle forces were then determined. The resultant hip joint contact and muscle forces were chosen from the frame where the highest hip contact force occurred, approximately at 45% of the walking cycle. Muscle forces were applied at a set of nodes on the femur outer surface, corresponding to the specific location of the muscle attachments. The contribution of every muscle as well as gravity forces were included. The hip load was applied at the top surface of the implant such that the line of action passed through the femur head centre. Regarding the boundary conditions, the model was constrained using physiological joint constraints, in which rigid body motion was prevented using displacement constraints on three nodes of the bone model mesh positioned on the lateral distal condyle and in the hip and knee joint [7] (Fig 4). Same loads and boundary conditions were used in each of the 256 models considered in this study, since the bone geometry and the position of the prosthetic hip does not vary between models.

**Fig 5:**
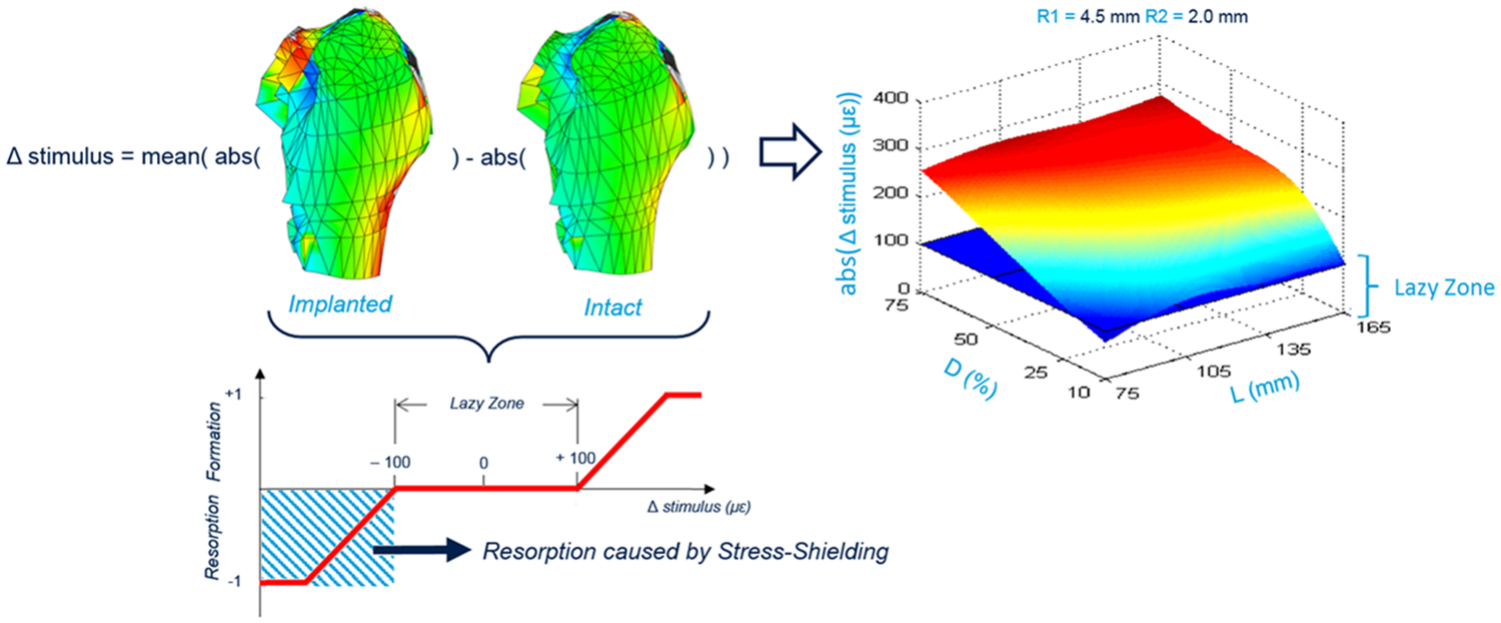
Δstimulus for the Gruen Zone I and a combination of R1 = 4.5 mm and R2 = 2mm plotted on a 3D surface. Representative plot of the 208 obtained data sets. The results were depicted using 3D surface plots where the z-axis represents the mean reduction of the strain value in the studied zone (12 sub-zones or Gruen Zone I) after the implantation and the other two axes describe the implant geometry, in this case, L and D. A threshold plane in blue color is depicted at 100 μstrains [44]. This plane represents the limits of the lazy zone, where neither resorption or formation is predicted. To include also the other two geometry parameters (the radiuses that compose the cross-section surface), it was plotted one graph for each combination of R1 and R2 values. In total, 208 3D surface plots (13 regions x 16 combinations of R1 and R2) were depicted. These 208 plots represent the input and desired output data used to train the MLT.

**Output of the FE analysis**: The stress shielding effect was evaluated as the difference between the maximum absolute principal strains detected in corresponding mesh elements of the implanted and intact bone models. The results were obtained by doing a simple arithmetical subtraction between the strain values detected in corresponding elements. Thereafter, the mean shielding effect in the region of interest, named Δ*stimulus*, was determined as the mean value of the strain reduction within the region and was used as indicator to quantify the stem performance. The lower this value, the more physiological the implant, i.e. the strains are more similar in the intact and implanted bones. The results were analyzed according to the system defined by Gruen et al. [43], which consists of dividing the femur areas around the implant in 7 regions of study. This study was focused on the Gruen Zone I, where greater stress shielding is expected. In addition, the elements of Gruen Zone I were further divided in 12 sub-zones to assess the local influence of the implant design on the femur strain distribution. In this way, 13 regions of each of the 256 implanted models were independently evaluated for the effect of the hip implant presence into the bone (Fig 5).

### 2.2.- Implant optimization by machine learning techniques

Two different MLTs were tested to solve the optimization problem: artificial neural networks (ANNs) and support vector machines (SVMs). Generally, SVM has greater updating capacity than ANN, because once the model is generated and presented with a new observation, if the model is unable to estimate correctly the value, it simply adds this observation to the support vectors set without the need of a new training loop [10]. However, for the ANN, new training of the whole network is needed in order to include a new observation [10,45].

Both MLTs were trained with the results obtained from the 3D FE parametric analysis. The Δstimulus was selected as output of the MLT since periprosthetic bone remodeling is highly associated with stress shielding. The goal of the analyses with MLTs was to find an optimized hip implant that leads to lack of bone resorption. The combination of inputs (geometrical parameters of the implant) and the output (strain reduction by the presence of the hip implant), for each of the 13 areas of study (12 sub-zones + Gruen Zone I), was used to create a data set. The training data set taught the MLT, which adapted itself by changing the weight vectors that characterize connections inside the structure. This process was iterative and the training task was repeated until convergence. To determine the accuracy of the technique, the absolute relative error (RE) and the correlation coefficient (RSQ) were calculated as follows:

$$RE=abs\left(\frac{\hat{\theta }-\theta }{\theta }\right)$$ (1)

$$RSQ=\frac{\sigma_{xy}}{\sigma x\;\sigma y}$$ (2)

Where $\hat{\theta }$ is the predicted Δstimulus, *θ* is the real Δstimulus, *σ*_*xy*_ is the covariance between predicted and real values, and *σ*_*x*_ and *σ*_*y*_ are the standard deviations. The RE shows the efficiency during the training process to find correlations between inputs and outputs. Regions with RE greater than 10% were not taken into account for the selection of the definitive optimal parameter values.

For the ANN technique, different networks, with different number of artificial neurons, were built and the RE calculated to determine the optimum number of neurons in the hidden layer. The SVM algorithm uses a kernel function that transforms the parameter input space into a feature space of larger dimension. To increment the accuracy of the training task, it is possible to choose the value of the kernel function parameters to get a more accurate input-output transfer function. The parameters that can be changed are the cost rate (*C*), that controls the tradeoff between achieving a low training error and a low RE during test testing error to generalize the classifier to unseen data; the margin (*ε*), or minimum distance between the hyperplane and the training set samples and the maximum acceptable variance (*σ*) for the output Gaussian noise. Many different options were tested for those parameters. The combination that returned the best performance, according to a low RE and computational speed, was chosen to train each of the 13 SVMs associated to each studied region. It is also important to limit the complexity of the network to prevent the overfitting problem, which occurs when the algorithm function is adapted too well to a specific training set and returns accurate desired outputs only with training set data but totally inaccurate values outside. Therefore, the relative error was evaluated during training to avoid possible overfitting. To evaluate the network accuracy a *k-fold Cross Validation* process was conducted. In this process, the data set was not totally used to train the network but was divided into 3 groups. One of them (*Training Set*) was used to train the network and the values from the other two groups were used to test the network (*Test Set*) and validate it (*Validation Set*). Then, the training process was performed k times (*k* = 8 for this study) for each network structure, shuffling the elements inside each group every time. In addition, different test set sizes were compared with the aim of investigating if the test size has an influence on the obtained errors.

Once the MLTs were built, a pattern-search minimization algorithm was used to get the optimal geometry, exploring new values of the input parameters. Pattern search is a family of numerical optimization methods that do not require the gradient of the problem to be optimized. Hence they can be used on functions that are not continuous or differentiable, such as the presented problem. The problem was formulated as minimization of a loss function (average difference of maximum absolute principal strain between implanted and healthy models).

**Fig 6:**
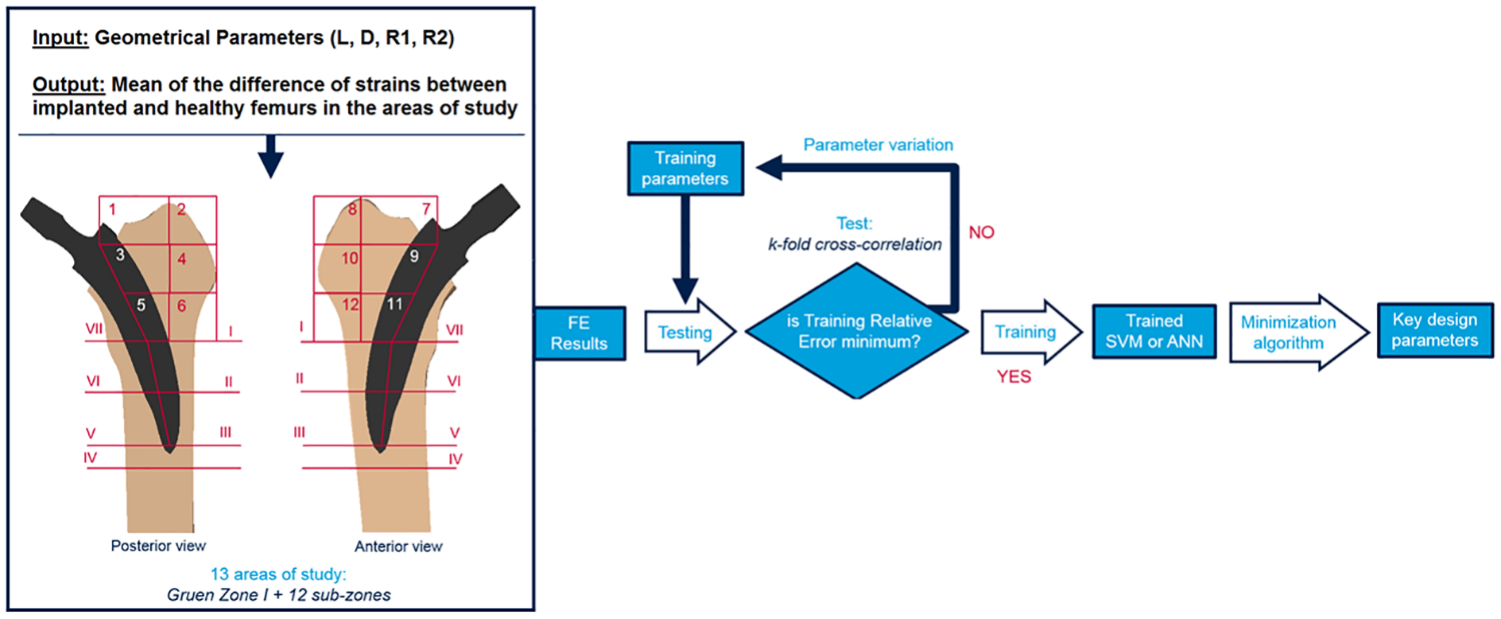
Flow chart of a typical training task using MLT. During the training, the error of the output prediction associated to the input parameters is minimized. Once the MLT is trained, the minimization algorithm find the best combination of design parameters to reduce the proximal stress shielding.

The optimization algorithm minimizes the function, exploring unseen values of the selected parameters of the hip implant geometry. The ranges of exploration for the 4 geometrical parameters were selected a priori according to the dimensions of the bone and considering a clinically admissible shape. These values were as follows; *L* (75–165) mm, *D* (10–90) %, R*1* (4–8), *R2* (1.5–4) mm. It should be noted that if the lowest limit for each parameter would not be fixed, probably the MLT would find null value for all parameters. The procedure is illustrated in the flow chart of Fig 6. This process was repeated for the 13 regions of study (12 sub-zones + Gruen Zone I).

Using the FE parametric analyses, the MLT and the search pattern algorithm, a set of optimal design parameter values were calculated for each of the 13 studied bone zones. Thereafter, a set of parameter values that characterize the optimal design for the overall study region was chosen based the results obtained in the different regions. Finally, once the key design parameter values were chosen, the performance of the selected combination of parameters was evaluated in all sub-zones and extended to the other Gruen Zones to assure that the optimized implant design reduces the stress shielding in the Gruen Zone I, but does not increase it in the remaining Gruen Zones. For this purpose, a new FE model was created with the optimal hip implant design to evaluate the stress shielding effect. In addition, the optimal implant geometry was compared with the original short-stem geometry (Nanos^®^ short stem, Smith & Nephew, Germany) to quantify the improvement or worsening of the new design in each Gruen Zone.

## 3.- Results

### 3.1- 3D FE parametric study

**Fig 7:**
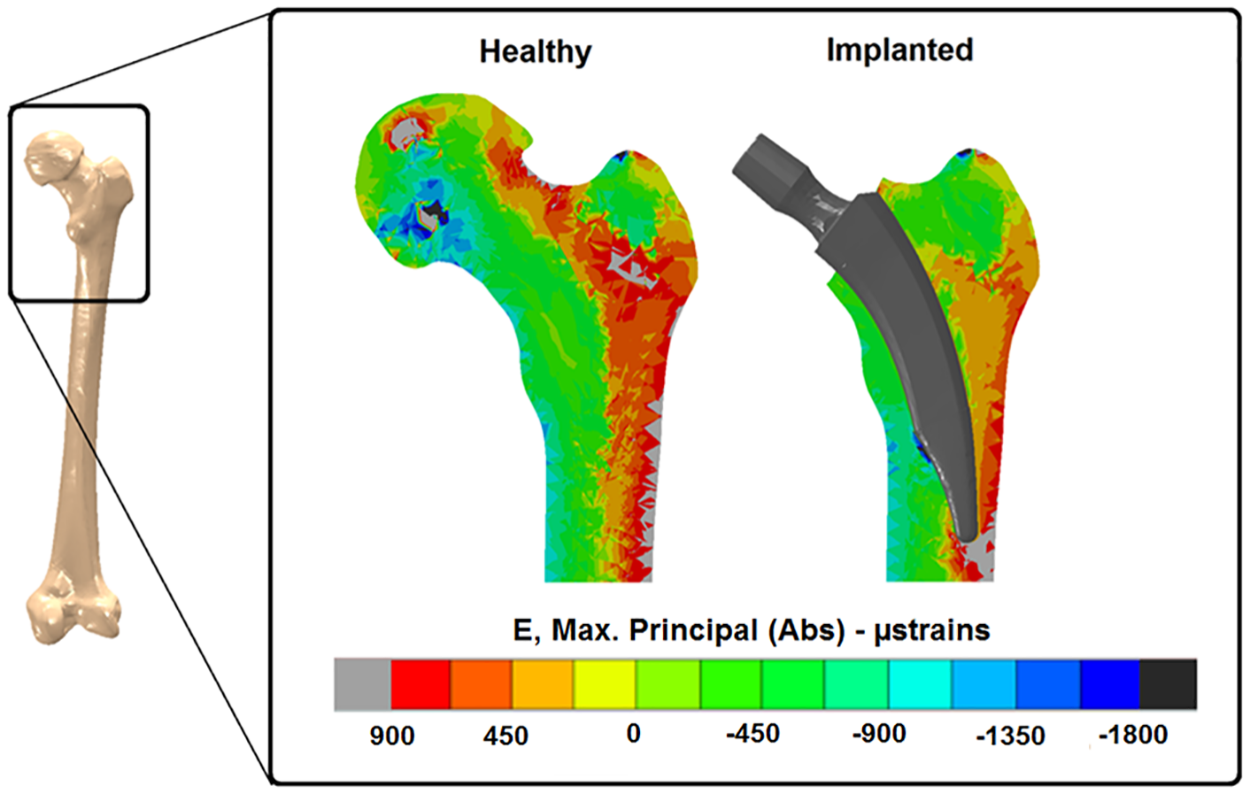
Maximum absolute principal strains at the mid-coronal cross section of the proximal part of the femur for intact and implanted model with the Nanos^®^ short stem.

All the models analysed showed some degree of stress shielding due to implant insertion. Fig 7 shows the stress shielding produced by the insertion of the original Nanos^®^ implant. A reduction of the strains in the proximal lateral aspect of the bone of up to 600 μstrain was determined (Gruen Zone I). In the medial aspect of the bone, a small region was determined where the strains in the implanted bone were higher than in the intact femur (Fig 7).

In addition, considering the Δstimulus obtained for the 256 models and for the 13 regions of study, the Δstimulus decreases as L and D decrease. However, the geometrical parameters R1 and R2 were not so influential in the reduction of the stress shielding, compared with the impact of the other two parameters. In addition, the length did not have a big influence if R1 was small.

### 3.2- Implant optimization

For the ANN technique, the most accurate training tasks were achieved when the number of neurons in the hidden layer were 9, 6, 20, 40, 20, 30, 40, 30, 30, 20, 8, 30 and 60 for sub-zones from 1 to 12 and for Gruen Zone I, respectively; pointing out the complexity of the problem for each of the studied zones. The relative errors during test using k-fold cross validation were lower than 10% for all studied zones, except for sub-zones 10, 11 and 12 (RE of 16.72%, 21.6% and 20.17%, respectively). For the SVM, RE during test were lower than 10% in all studied areas, except for the sub-zones 10 and 11 with RE of 13.43% and 18.6%, respectively. For all studied sub-zones and Gruen Zone I, test RE were lower for the SVM than for the ANN. Using both MLTs, the correlation coefficient (RSQ) was very close to 1 (0.9998).

**Fig 8:**
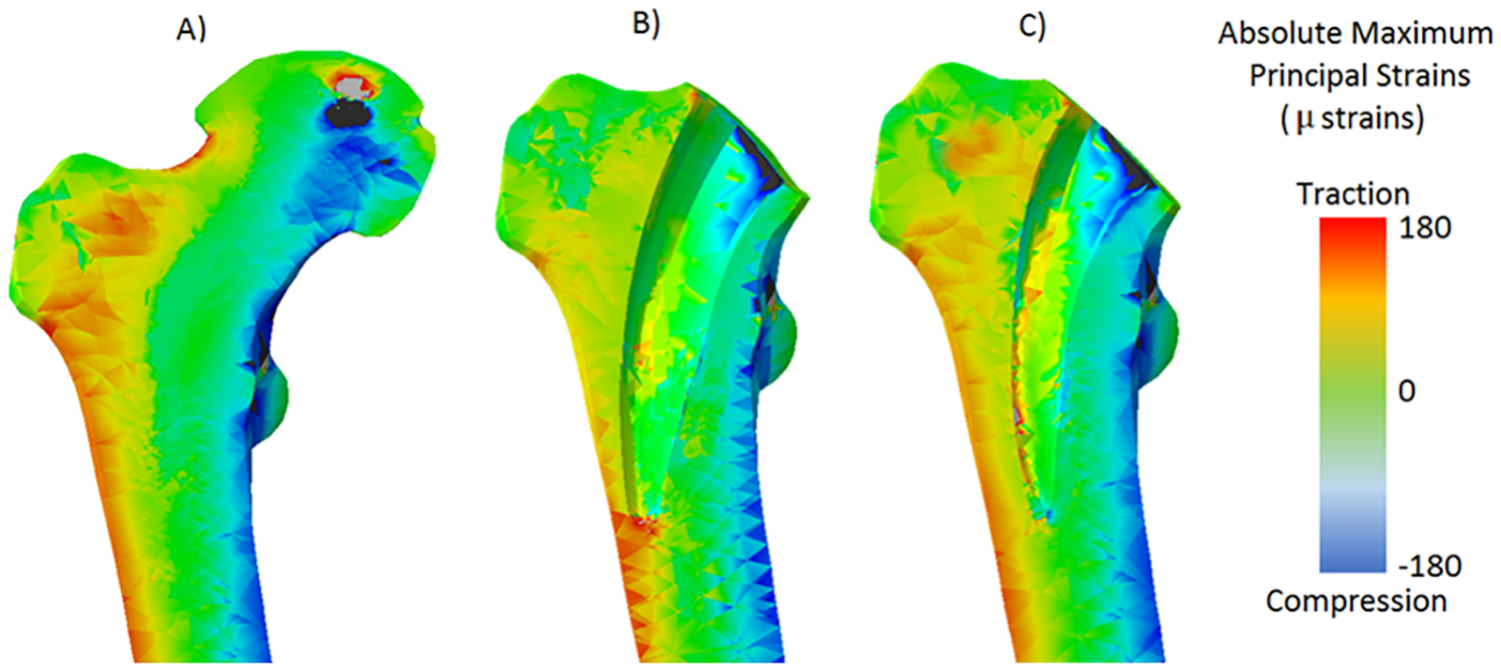
Comparison of the absolute maximum principal strain distribution between intact bone model (a), the one implanted with the original stem (b) and the new design (c).

The pattern-search minimization algorithm followed similar trends for both the ANN and SVM techniques, however, since lower REs were predicted in SVM compared with ANN, the optimized parameters were chosen according to the SVM results. Moreover, for the sake of simplicity, only the results of the SVM are shown. SVM combined with the minimization algorithm led to similar optimal values for all geometric parameters in several sub-zones (3, 6, 7, 9, 12 and in the whole Gruen Zone I). The optimal parameter values for these regions were: L = 90 mm; D = 36%; R1 = 4 mm; R2 = 1.5 mm. Therefore, these values were taken as key design parameters for the optimized implant design. The optimized implant design showed a reduction in stress shielding compared with the original implant design, however some degree of stress shielding was still present (Fig 8).

**Fig 9:**
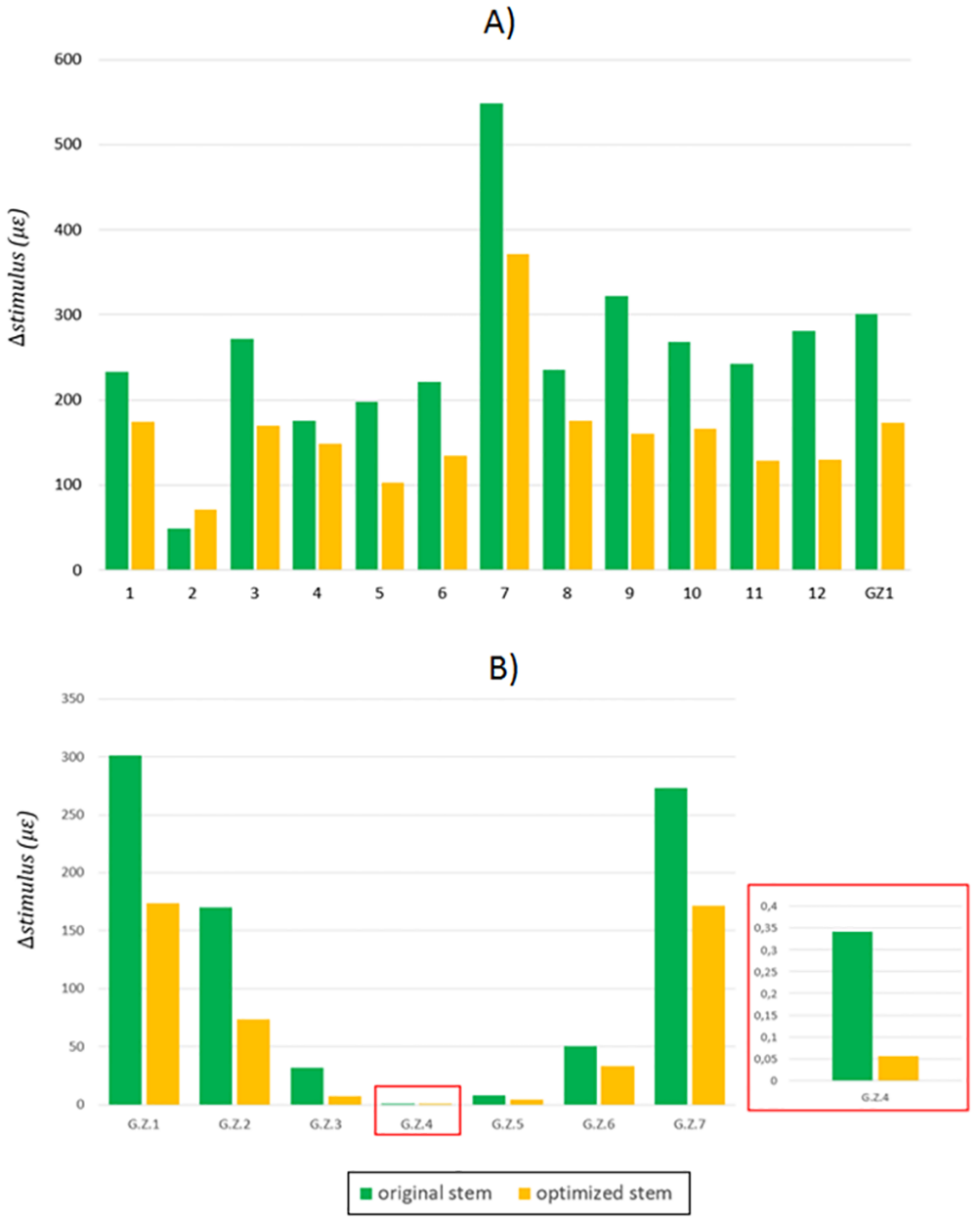
Δstimulus evaluated in the original and optimized stem. Comparison done between the sub-zones of the Gruen Zone I (a) and all Gruen Zones (b).

The optimized hip implant design reduced the stress shielding in all the considered sub-zones and Gruen Zones, except for sub-zone 2 (Fig 9). However, the Δstimulus in this area was lower than 100 μstrains, and therefore, it is included in the lazy zone. Reduction of strains up to180 μstrains were found in the sub-zone 7 within Gruen Zone I (Fig 9). On the other hand, the Δstimulus in sub-zone 5 decreased from 195 μstrains to 100 μstrains. Regarding the remaining Gruen Zones, the reduction of Δstimulus using the new optimized implant design was always positive for all Gruen zones. The reductions of strains were 130 μstrains for Gruen Zone I, 100 μstrains for Gruen Zone II and VII and lower than 100 μstrains in the rest of the Gruen Zones.

## 4.- Discussion

In this study, we investigated whether the geometry of a short stem hip implant can be further optimized to reduce stress shielding at the proximal femur. For that purpose, Finite Element (FE) analyses were combined with Machine Learning Techniques (MLT) and search pattern minimization algorithms. These techniques have been previously used to solve different problems in biomechanics, such as the prediction of the proximal femoral loads based on bone morphology [23] or the prediction of the atheroma plaque rupture [20]. However, the potential of this technique to optimize joint implants has never been investigated before. Here, we show that the combination of these three techniques has the potential to optimize a joint implant towards reduced stress shielding.

The combination of FE analyses, MLTs and search pattern optimization algorithms presents an important advantage in terms of computational costs. The finite element method for the analysis of such complex models requires long computational time. However, the simulation time can be significantly reduced combining finite element with machine learning techniques. For the ANN and SVM, the computation training time was 6 ± 3 and 4 ± 2 min, respectively (once the optimal parameters had been chosen by k-fold cross validation), and the time of response when a new case is evaluated was negligible since ANN and SVM techniques only evaluate a function, providing an immediate estimated response. However, the computational cost to build and simulate each finite element model is 9 ± 3 h. Moreover, MLTs allow to explore a continuous range of values for a set of parameters, rather than a discrete one. In addition, these algorithms determine the desired output for a set of unseen parameters, minimizing the number of FE models needed to find an optimized implant design. Furthermore, we compared the performance of both MLTs: Support Vector Machine (SVM) and Artificial Neural Network (ANN). For both MLTs, the correlation coefficient (RSQ) was very close to 1, pointing out that the model has been well trained. Although, it was shown that both techniques represent a powerful tool to predict the behaviour of an implanted femur in terms of stress shielding quantification, lower degree of accuracy was achieved with the ANN since the obtained relative errors were greater for all studied subzones. The nature of the dataset at hand determines which MLT works best; therefore, different MLT algorithms need to be check for each specific application [10].

FE models were used to investigate how the strain distribution varies in the proximal region of the femur due to changes in hip implant geometry. 256 hip implant geometries were created based on the Nanos^®^ short stem implant. The total stem length (*L*), the stem thickness evaluated at the most distal cross-section in contact with the bone (*R1* in the lateral side, *R2* in the medial side) and the distance between the neck and the same cross-section (*D*) were selected as main geometrical parameters of the hip implant. All implanted models were compared with its corresponding healthy bone in to evaluate the degree of stress shielding created by each implant design. The results obtained in the FE analyses were used to build an artificial neural network and support vector machine, comparing both machine learning techniques. The machine learning algorithm predicts the mean of the difference of maximum absolute principal strains between the implanted and healthy models for a specific region based on a set of input parameters. Finally, machine learning was combined with a search pattern minimization algorithm to find a set of key input parameters that result in an implant design that produces the minimum stress shielding.

Our results show that to optimize the implant design towards reduced stress shielding, the tendency is to decrease its stem length (L) and to reduce the length of the surface in contact with the bone (D). The two radiuses (R1 and R2) that characterize the stem width at the distal cross-section in contact with the bone were less influential in the reduction of stress shielding compared with the other two parameters; but they also play a role and thinner stems present better results. Interestingly, the total stem length did not influence the strain distribution if R1 was small. The implant performance should be observed as a consequence, not only of the stem length, but as a combination of the four considered parameters. Considering the results of the SVM combined with the optimization algorithm, the chosen key design parameters for the new optimized implant design were: L = 90 mm; D = 36%; R1 = 4 mm; R2 = 1.5. Considering that the original short stem had dimensions of (L = 92 mm, D = 60%, R1 = 4.2 mm and R2 = 2.9 mm), it could be concluded that short stems can be designed to minimize stress shielding by reducing the stem length (L = 90 mm), creating a contact surface cross-section positioned proximally (D = 36%) and reducing the stem thickness (R1 = 4 mm, R2 = 1.5 mm). The optimized implant presents a small radius compared to the original implant (R2 optimized: 1.5, R2 original: 2.9). This could give the impression of a very thin implant in the optimized condition; however, R1 and R2 are related to the parameter D. R1 and R2 indicate the width of the hip implant at the section indicated by D, which in the optimized implant is located more proximal than in the original implant. In the optimized implant, R1 and R2 at the section located at 60% of the implant length (D of the original implant), are close to the value of the original implant (S1 Fig). The selected optimal values obtained with the minimization algorithm reduced the stress shielding effect for all studied sub-zones and Gruen Zones, except for sub-zone 2. However, the Δstimulus for this zone was lower than 100 μstrains, and therefore, remodeling is not expected (values lower than 100 μstrains belongs to the lazy zone). On the other hand, the Δstimulus in sub-zone 5 decreased from 195 μstrains to 100 μstrains, turning this area stable. It should be pointed out that the optimal values for the studied geometrical parameters were different for each one of the analyzed zones (12 sub-zones and the Gruen Zone I), showing that does not exist a specific combination of parameters that fulfils the aim of minimum stress shielding for all the zones at the same time.

Only few studies have investigated implant optimization to reduce the bone remodeling signal and the influence of hip implant design parameters on stress shielding [27,46–49]. Moreover, all of them are related to long stem implants. In this study, the optimal performance was found using a hip implant with a total length smaller than 120 mm; confirming the potential advantages of the short stems [50]. For the parameter characterizing the implant length in contact with the bone (D), optimal designs resulted in values smaller than 50%. This means that the optimal position for the cross section that separates the stem into a part which is directly in contact to the cortical bone and a free part is closer to the implant neck than to the distal tip. A small value for the D parameter results in a reduction of the thickness in the distal part of the implant, where the stem decreases its width until the distal tip. The lateral sub-zones of the Gruen Zone I were less influenced by the shielding effect for implants with a shorter stem-contact-surface internal length (D = 10%). In the medial side of Gruen Zone I, the optimal model D parameter was estimated to be around D = 36%. In this case, the cross-section is in the thicker part of the proximal femur, surrounded by the greater trochanter bone tissue. Positioning the cross-section in the thicker part of the femur could improve the stability of the implant, which is fitted and kept in position by a large quantity of trabecular tissue. This is in agreement with the study by Chang et al. [27], who minimized the difference between the strain energy density of the intact femur and an implanted bone using an implant with thin mid-stem diameter and a short stabilizing distal tip, concluding that a thin mid-stem diameter with a short stabilizing distal tip minimizes the bone remodelling signal while maintaining satisfactory stability. The D parameter is not the only one that influences stem thickness; also, the cross-sectional area plays a role. Varying the parameters R1 and R2 is possible to modify this cross-sectional area, however the results did not show a clear trend on how they contribute to implant optimization. Interestingly, Tanino et al., [47] in their optimization study of a stem using adaptive p-method, found also that the medial width of the midcross-section did not follow a clear trend in its influence on the hip implant performance. In addition, the obtained results for R1 and R2 were similar to those found when optimizing implants to reduce the stresses in the cement layer [48,49], where a narrower optimal stem shape at the proximal side was found. Other studies related to the optimization of hip implants include the effect of other geometrical parameters such as the head diameter and the neck angle [51], dimensions of different cross sections along the whole stem [28,30,52] or presence or absence of medial collar [53]. To the best of the authors’ knowledge, this is the first study where the potential of MLTs for hip implant optimization was proven.

Some remaining limitations of this study need to be stated and considered. First, normal walking was considered as the applied load to the FE model of the hip joint. However, other demanding activities, such as stair climbing, could play an important role in the performance of a hip implant and it should be investigated in the future. Second, a larger group of parameters could be included. However, the needed models to feed the MLT exponentially increases as the number of parameters increases. For example, the shape of the implant cross-section has a significant effect on the maximum tension and compression generated within the bone [53]. It could be interesting to design different cross-sections, at different positions along the implant stem, and evaluate the optimal shape for each of them [48,52]. Third, the procedure can also be extended to a larger group of femur geometries, testing the implants with bone models obtained from different patients. However, although absolute strain levels in another human femur geometry may vary somewhat from those found in this study; this is expected to have little influence on relative differences between the healthy and implanted models. Weinans et al. [54] concluded that although the choice of input parameters of FE models can substantially affect stress shielding in an individual, this choice had virtually no effect on the relative differences in femoral periprosthetic stress shielding between individuals. Fourth, despite the hip implant position was supervised by experts of our clinic, small changes in the hip implant position may produce slightly different strain patterns. Parameters related to surgical position of the implant inside the bone could also be studied to optimize the positioning of the stem during the THA performance. In this study, we did not proof the validity of the findings in vivo. The next step should be to investigate if the optimized design leads to reduced bone resorption in patients.

In conclusion, the design of short stem hip implants can be further optimized to reduce stress shielding. Implants should be design with a small stem length (L) and a reduced length of the surface in contact with the bone (D). Regarding, the width of the hip implant, a clear tendency was not found. Finally, it can be concluded that the optimization approach based on a combination of FE and MLT offers new and innovative possibilities for the design of hip implants never explored before. These analyses can help in the design of new prosthesis and also in the decision-making of surgeons when choosing the most adequate implant.

## Supporting information

S1 Fig Geometry of original and optimized implants. (TIF)
