# Supplementary figures and images for: Machine learning techniques for the optimization of joint replacements: Application to a short-stem hip implant
Source: PLoS One. 2017 Sep 5;12(9):e0183755. doi: 10.1371/journal.pone.0183755 (PMC5584793; doi:10.1371/journal.pone.0183755)

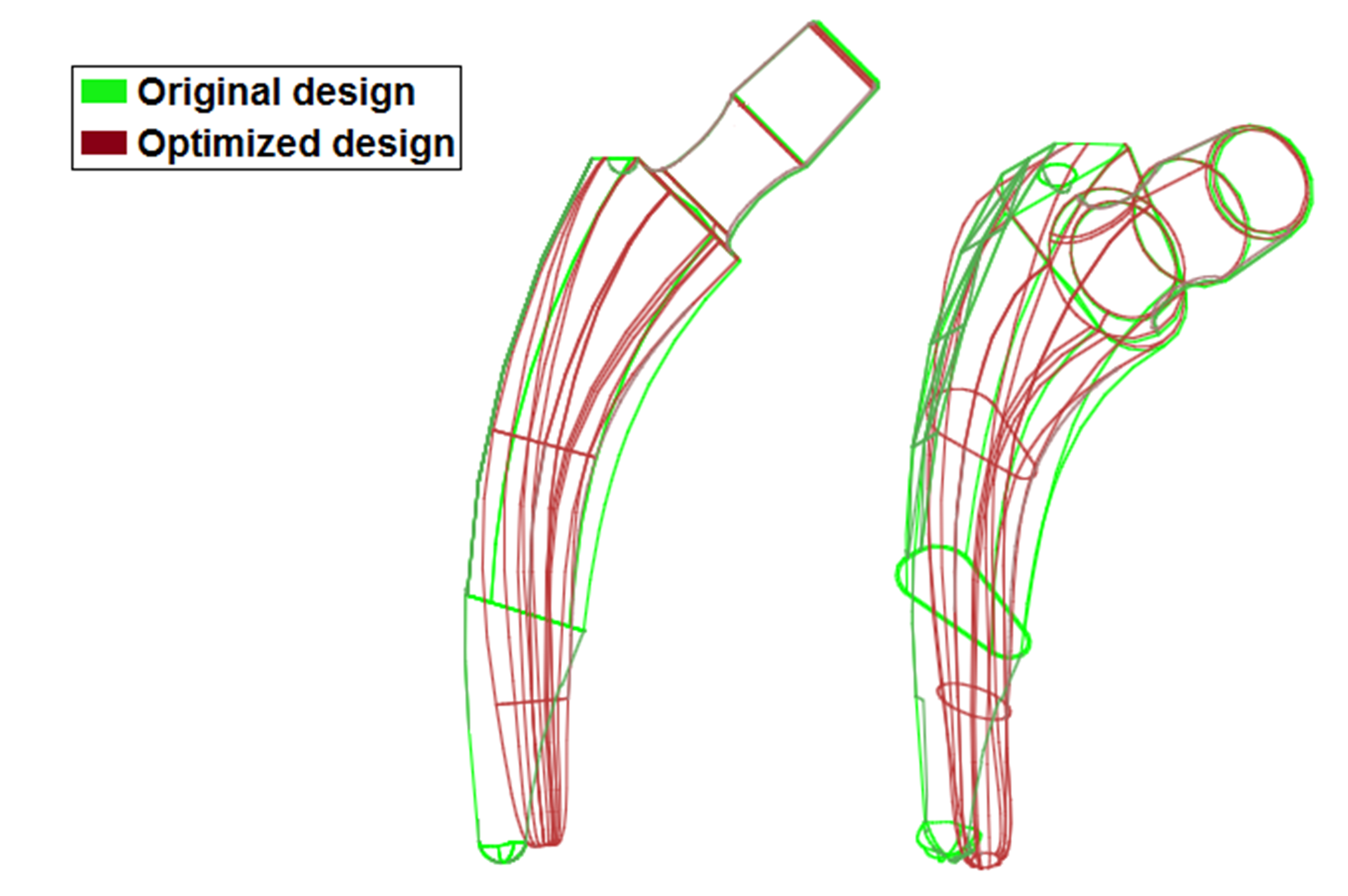

Supplement: S1 Fig — (TIF) [file pone.0183755.s001.tif]
